# Ultrahigh Ni‐Rich (90%) Layered Oxide‐Based Cathode Active Materials: The Advantages of Tungsten (W) Incorporation in the Precursor Cathode Active Material

**DOI:** 10.1002/smsc.202400135

**Published:** 2024-07-10

**Authors:** Marcel Heidbüchel, Aurora Gomez‐Martin, Lars Frankenstein, Ardavan Makvandi, Martin Peterlechner, Gerhard Wilde, Martin Winter, Johannes Kasnatscheew

**Affiliations:** ^1^ MEET Battery Research Center Institute of Physical Chemistry University of Münster Corrensstr. 46 48149 Münster Germany; ^2^ Institute of Materials Physics University of Münster Wilhelm‐Klemm‐Str.10 48149 Münster Germany; ^3^ Helmholtz Institute Münster IEK‐12 Forschungszentrum Jülich GmbH Corrensstr. 46 48149 Münster Germany

**Keywords:** precursor cathode active material, primary particles, sol–gel coating, ultrahigh Ni‐rich layered oxides, W modification

## Abstract

Minor amounts of tungsten (W) are well known to improve Ni‐rich layered oxide‐based cathode active materials (CAMs) for Li ion batteries. Herein, W impacts are validated and compared for varied concentrations and incorporation routes in aqueous media for LiNi_0.90_Co_0.06_Mn_0.04_O_2_ (NCM90‐6‐4), either via modification of a precursor Ni_*x*_Co_*y*_Mn_*z*_(OH)_2_ (pCAM) within a sol–gel reaction or directly during synthesis, i.e., either via an W‐based educt or during co‐precipitation in a continuously operated Couette–Taylor reactor. In particular, the sol–gel modification is shown to be beneficial and reveals >500 cycles for ≈80% state‐of‐health NCM90‐6‐4||graphite cells. It can be related to homogeneously W‐modified surface as well as smaller and elongated primary particles, whereas the latter are suggested to better compensate anisotropic lattice stress and decrease amount of microcracks, consequently minimizing further rise in surface area and the accompanied failure cascades (e.g., phase changes, metal dissolution, and crosstalk). Moreover, the different incorporation routes are shown to reveal different outcomes and demonstrate the complexity and sensitivity of W incorporation.

## Graphical abstract

LiNi_0.90_Co_0.06_Mn_0.04_O_2_ (NCM90‐6‐4), an ultrahigh Ni‐rich material, reveals high specific capacity/energy but suffers from rapid capacity/energy fade. Among a variety of possible modifications, W‐based coatings are literature‐known to be promising. Herein, this coating is directly applied in water on the hydroxide‐based NCM precursor via sol–gel approach allowing better particle coverage and primary particle modifications, finally boosting the cycle life.

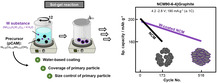

## 1 Introduction

Layered oxide‐based Ni‐rich cathode active materials (CAMs) with a Ni content even above 80%, e.g., LiNi_0.90_Co_0.06_Mn_0.04_O_2_ (NCM90‐6‐4), are promising in the field of electric mobility due to even higher gravimetric/volumetric energy/power at comparatively lower costs.^[^ ^1^, ^2^ ^]^ However, material synthesis, surface sensitivity, thermal stability, safety aspects, and cycle life remain a challenge.^[^ ^3^ ^]^

Several approaches are literature‐known, including surface modifications, e.g., via doping, coating, or structural design, e.g., via single crystal or core shell,^[^ ^4^, ^5^, ^6^ ^]^ whereby W addition is particular promising because of its simple processing and incorporation paths, i.e., either directly on NCM via coating^[^ ^7^, ^8^ ^]^ or during NCM synthesis, either via doping during calcination step^[^ ^9^, ^10^ ^]^ or via modification of precursor Ni_*x*_Co_*y*_Mn_*z*_(OH)_2_ (pCAM) during/after co‐precipitation.^[^ ^11^, ^12^, ^13^ ^]^

The beneficial effect of W is attributed to strain suppression during the H2/H3 phase transformation, which in turn can minimize microcracks and contact losses, thus preventing further rises in surface area, transition metal dissolution, and electrode crosstalk.^[^ ^12^, ^14^ ^]^ Additionally, W‐containing species tend to migrate toward surface to form Li_*x*_W_*y*_O_*z*_ and Li_4+*x*_Ni_1−*x*_WO_6_ phases, within calcination step,^[^ ^15^, ^16^ ^]^ and are claimed to suppress side reactions of highly charged Ni with inactive materials, e.g., electrolyte.^[^ ^7^, ^17^ ^]^ Finally, W‐based coatings lead to smaller, elongated primary particles likely via agglomeration of W in the grain boundaries and suppress their further growth during calcination,^[^ ^11^ ^]^ which might affect performance characteristics via size and morphology and pave way for additional CAM design control.^[^ ^14^, ^18^, ^19^, ^20^, ^21^ ^]^ Besides these benefits, modifications on hydroxide‐based pCAMs, which are not water‐sensitive, can be done in aqueous solutions.^[^ ^7^, ^8^ ^]^

**Figure 1:**
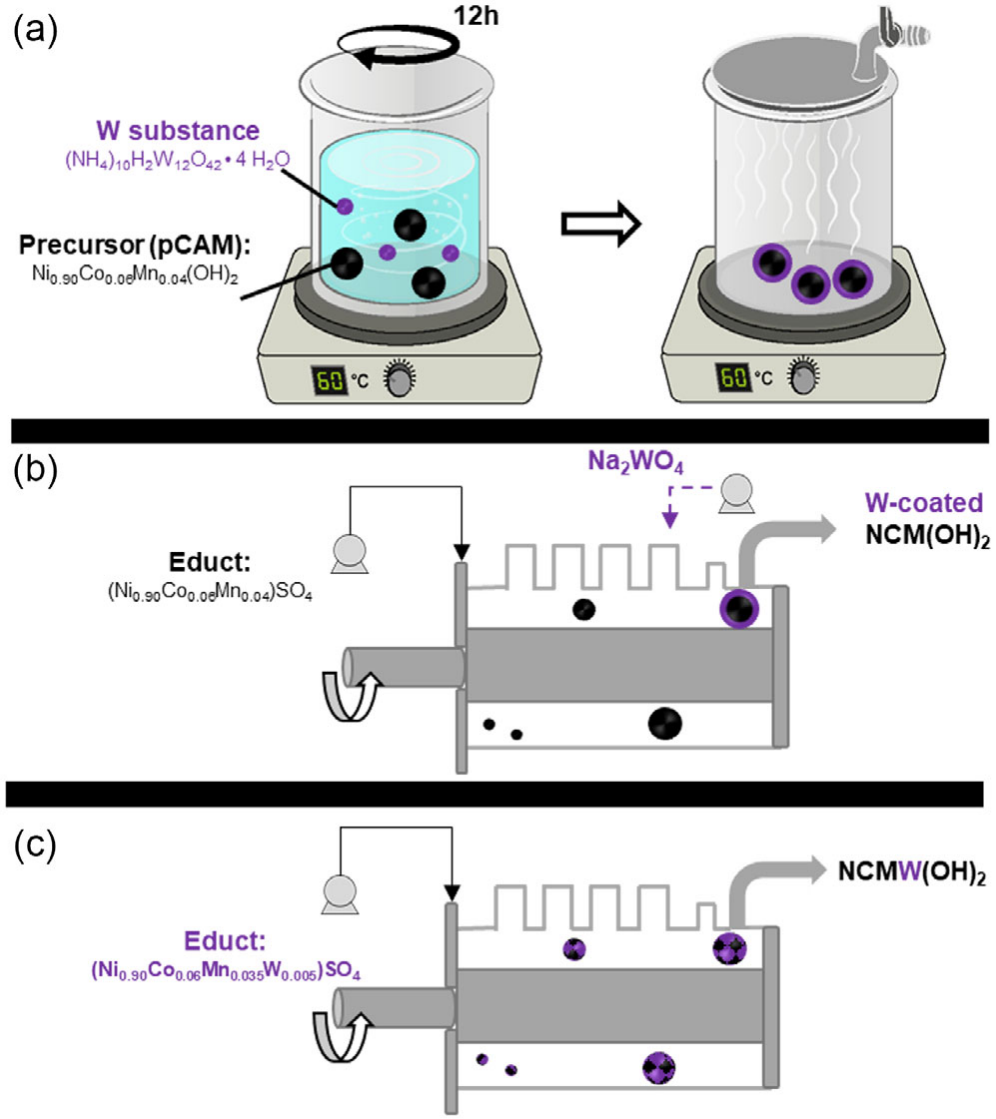
Schematic illustration of different W modifications of pCAM; either as a coating via a) sol–gel reaction, b) coating at the end of co‐precipitation, or c) W incorporation during pCAM co‐precipitation with W‐based educts.

In this work, pragmatic and application‐relevant, i.e., water‐based W doping routes of pCAMs are varied and validated for NCM90‐6‐4, i.e., ultrahigh Ni NCM. Either, different W concentrations are coated via sol–gel reaction on pCAM (**Figure** **1**a) or incorporated during pCAM synthesis at final stage of co‐precipitation within continuously operated Couette–Taylor reactor (CTR), shown in Figure 1b, or directly incorporated via educts (Figure 1c). Sol–gel reactions are well known for their simplicity and exact control of nano‐ as well as micro‐sized structures. They are based on a colloidal solution (Sol), which agglomerates into a gel‐like polymer covering the precursor.^[^ ^22^ ^]^ The CTR, a recently established technology for synthesis of spherical‐shaped particles, offers higher mixing intensity, shortened reaction times compared to a continuous stirred tank reactor,^[^ ^23^ ^]^ and the possibility to encapsulate in situ the formed particles with different coatings via different ports, thus realizing pragmatic technical possibilities to adjust and develop parameter for co‐precipitation.^[^ ^24^, ^25^ ^]^ Direct coating of pCAM at the end of co‐precipitation or via educt addition (Figure 1b,c) can be more efficient as it circumvents the (additional) coating processing step, e.g., within sol–gel, and can be theoretically more efficient. However, this work demonstrates their practical challenges, pointing to sensitivity and complexity of W incorporation.

## 2 Results and Discussion

In literature,^[^ ^7^ ^]^ ammonium tungstate ((NH_4_)_10_(H_2_W_12_O_42_)·4H_2_O) is coated on CAM, while in this work it is coated on the commercial precursor Ni_0.90_Co_0.06_Mn_0.04_(OH)_2_ (pCAM) either via sol–gel reaction (Figure 1a) or during pCAM co‐precipitation (Figure 1b) in CTR. In parallel, W‐modified pCAMs are also synthesized directly with W‐containing educts (Ni_0.90_Co_0.06_Mn_0.0350_W_0.005_)SO_4_ (Figure 1c). The final CAMs are obtained after annealing with lithiation (=calcination) and further labeled depending on W concentration and preparation routes. The CAMs prepared from commercial pCAMs and coated via sol–gel reaction are referred as 1) 0%_W, 2) 0.5%_W, and 3) 1%_W, and the CAMs originating from CTR are referred as 1) 0%_W_CTR, 2) 0.5%_W_CTR_Shell, and 3) W‐containing educts 0.5%_W_CTR.

**Figure 2:**
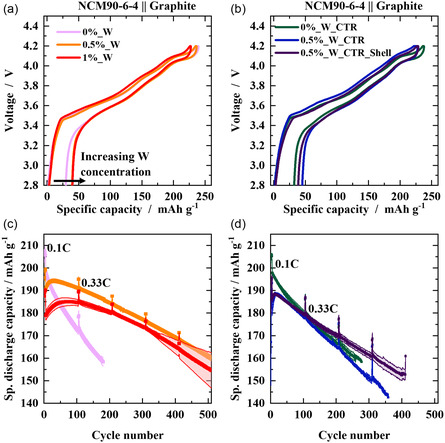
Galvanostatic charge–discharge data in NCM||graphite cells for different NCMs. Initial voltage profile of CAMs based on a) sol–gel coating b) modified in CTR. The respective charge–discharge cycling is depicted in c,d) at 0.33 C (1 C = 190 mA g^−1^). Standard deviation is calculated from at least three different cells. Cell voltage range: 4.2–2.8 V, N/P‐ratio: 1.15:1.00, electrolyte: 1 M LiPF_6_ in 3:7 vol% EC/EMC + 2 wt% VC.

The different CAMs are evaluated in NCM||Li (Figure S1, Supporting Information) and NCM||graphite cells (**Figure** **2**). The addition of W via sol–gel approach decreases the initial capacity proportional to W content and decreases initial coulombic efficiency (C_eff_) from ≈89% to ≈82% (Figure S1a, Supporting Information), similar to the approach for W addition via co‐precipitation (Figure S1b, Supporting Information). At harsher conditions, i.e., at enhanced upper cutoff voltages (UCVs) up to 4.5 V, the beneficial effect of W is even more pronounced as seen in Figure S1c, Supporting Information, in particular for sol–gel incorporated W.

Similar to NCM||Li metal, also in NCM||graphite cells the initial discharge capacities and initial C_eff_ (Figure 2a,b) decrease with increased W content. Nevertheless, the modification with W via sol–gel approach enhances capacity retention (Figure 2c). The limit of 80% state‐of‐health (SoH), an end‐of‐life criterium, is above 500 cycles for W‐based NCMs, while only 200 cycles for reference NCM. These effects are similar, but less pronounced for the modifications during co‐precipitation and W‐based educts, as seen in Figure 2d. The average discharge voltage and the difference between the average charge and average discharge voltage (voltage hysteresis) indicate the progress in cell resistance during charge–discharge cycling.^[^ ^23^ ^]^ The W‐containing electrodes via sol–gel method have the lowest growth in resistance (Figure S2a,b, Supporting Information) as well as a highest and most stable mean discharge voltage (≈3.7 V) and consequently reveal highest specific discharge energies and cycle life (Figure S3, Supporting Information). Though, the modification of W during co‐precipitation also improves the specific energy and stability, the influence is less pronounced (Figure S3, Supporting Information), which suggest other parameter than chemical composition to be crucial, likely particle‐related properties.

**Figure 3:**
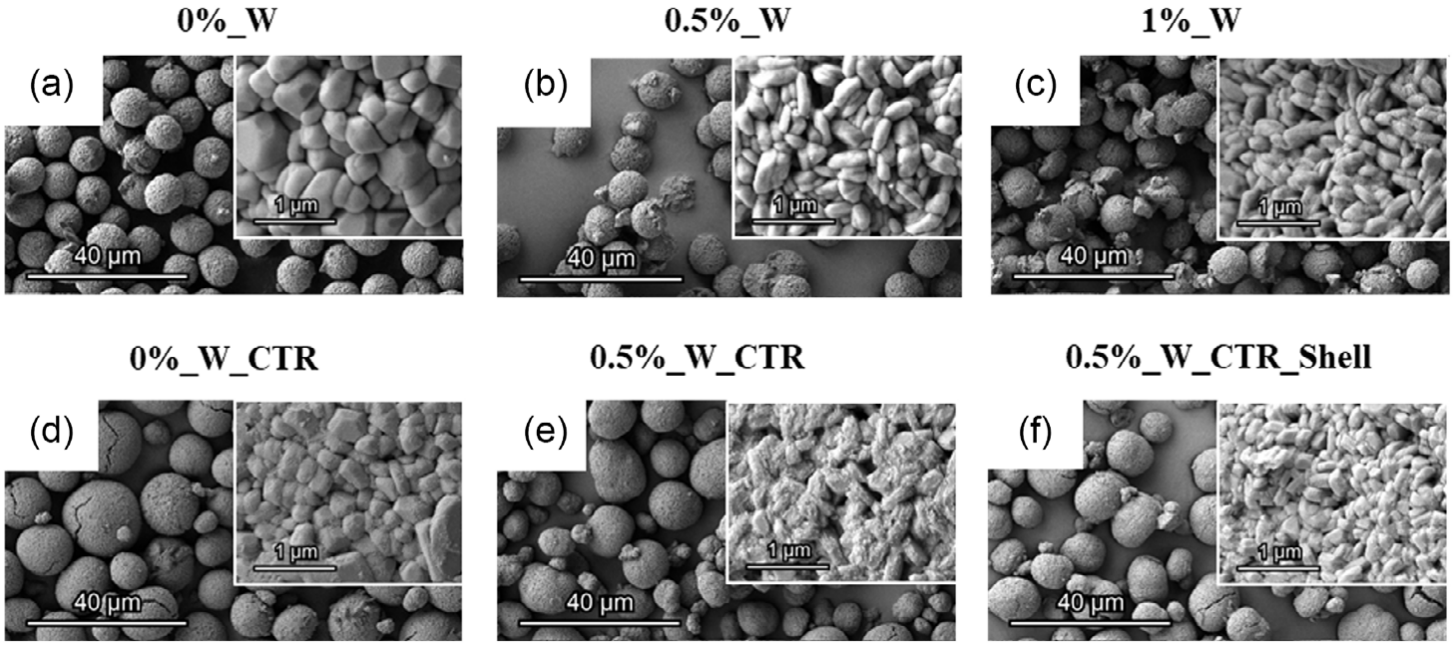
SEM images of different NCMs as defined and respectively entitled above respective images from (a–f). W‐containing Ni‐rich NCM have smaller and elongated primary particles. The secondary particle size of synthesized CAMs is larger than for the commercial CAM.

**Figure** **3** shows scanning electron microscope (SEM) images of the CAMs. The NCMs from commercial pCAMs, and sol–gel incorporated 0.5% and 1.0% W, depicted in Figure 3a–c, consist of micron‐sized (≈10 μm) spherical‐shaped secondary particles. The reference 0%_W has nano‐sized grain‐like primary particles (300–500 nm), which form a smooth surface (Figure 3a). With increasing W content, these primary particles get smaller, elongated, and closer packed (Figure 3b,c). Furthermore, some secondary particles are collapsed with enhanced agglomeration of fragments.

In contrast, the synthesized CAMs with a CTR consist of small (<5 μm) and large (>10 μm) secondary particles, which are spherical‐shaped (Figure 3d). Particles with a diameter >20 μm cannot maintain the spherical morphology, leading to formation of cracks. Similar to the 0%_W reference, the surface consists of grain‐like primary particles. W‐containing NCM incorporated via educts during precipitation tend to have less crack formation in the secondary particle (Figure 3e). The corresponding primary particles are smaller, but with rougher surface. The W‐containing NCM incorporated during precipitation (0.5%_W_CTR_Shell) has cracks (Figure 3f) and is similar to W‐free NCM (Figure 3d).

Focused ion beam (FIB) cutting proves the W‐induced changes of primary particles (Figure S4, Supporting Information). With increasing W concentration, the primary particles decrease in size and can be therefore packed more efficiently with less remaining voids between the primary particles (Figure S4a–f, Supporting Information). Primary particles of self‐synthesized CAMs (Figure S4g–l, Supporting Information) decrease also in size, leading to particles with less intergranular voids for 0.5%_W_CTR (Figure S4i,j, Supporting Information). However, the 0.5%_W_CTR_Shell materials shows inner porosities, which probably stem from altered reaction condition during precipitation, which affects the particle growth mechanism, thus demonstrating the complexity of coating R&D, as coating effectiveness sensitively depends from exact material consistency, i.e., previous material synthesis and treatment.

**Figure 4:**
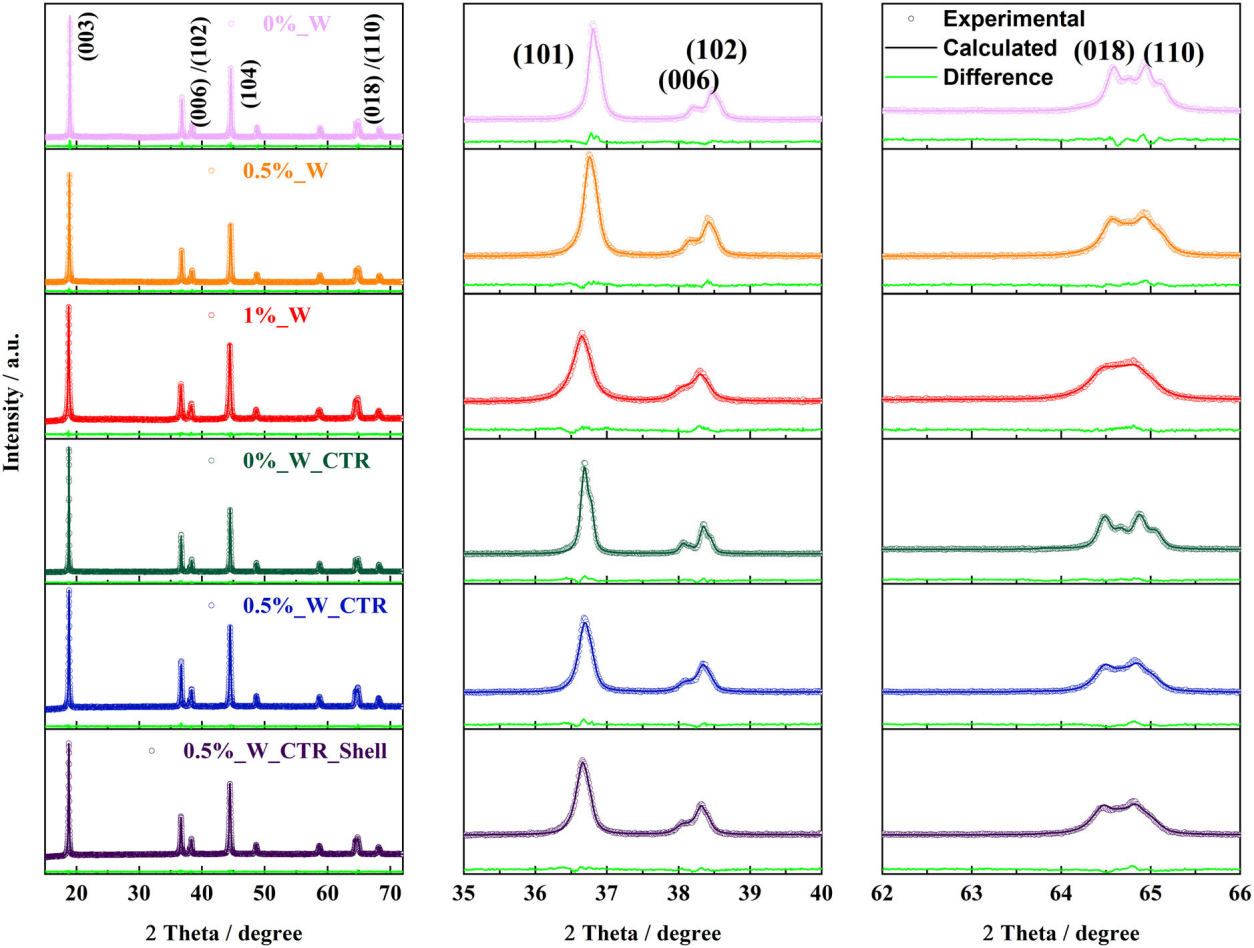
XRD patterns and Rietveld refinements of pristine and W‐containing Ni‐rich CAMs. Decreased sharpness of 018/110 peak indicates a decrease of atomic order.

X‐ray diffraction (XRD) of the respective CAMs is depicted in **Figure** **4**. Corresponding Rietveld refinements information is summarized in **Table** **1**. All samples have a phase‐pure hexagonal α‐NaFeO_2_ structure, which belongs to the $R\bar{3}m$ space group. The absence of impurity peaks or amorphous phases between 20° and 25° excludes crystalline Li_*x*_W_*y*_O_*z*_ phases likely due to the limit of detection for low W concentration.^[^ ^11^ ^]^ The splitting of (006)/(102) and (018)/(110) reflexes indicates high crystallinity and a good hexagonal ordering for the W‐free CAMs,^[^ ^26^ ^]^ while less peak splitting for W‐containing samples indicates a decrease in the atomic order, likely caused by W doping in the lattice.^[^ ^27^ ^]^ This trend is even more pronounced for 1% W.

**Table 1:** Listed values for D_50_, stoichiometry, BET surface area, and Li/Ni mixing degree for the NCMs.

CAM	D_50_ [μm]	Stoichiometry	BET surface area [m^2^ g^−1^]	Li/Ni mixing in%
0%_W	9.58	Li[Ni_0.903_Co_0.059_Mn_0.038_]O_2_	0.34	2.767
0.5%_W	10.09	Li[Ni_0.902_Co_0.059_Mn_0.038_W_0.001_]O_2_	0.53	3.496
1%_W	8.87	Li[Ni_0.901_Co_0.059_Mn_0.038_W_0.002_]O_2_	0.43	6.333
0%_W_CTR	14.38	Li[Ni_0.901_Co_0.060_Mn_0.039_]O_2_	0.22	3.226
0.5%_W_CTR	11.81	Li[Ni_0.903_Co_0.060_Mn_0.035_W_0.002_]O_2_	0.25	5.673
0.5%_W_CTR_Shell	11.97	Li[Ni_0.895_Co_0.061_Mn_0.042_W_0.002_]O_2_	0.30	6.076

The particle size, stoichiometry, surface area, and Li/Ni mixing of the different CAMs are summarized in Table 1. The CAMs based on the commercially available precursor (sol–gel NCM) and from CTR have a D_50_ value of 10 and 14.28 μm, respectively. However, the addition of 1% W cracks the secondary particles (see Figure 3c) and decreases particle size. The stoichiometries, obtained by inductively coupled plasma optical emission spectroscopy (ICP‐OES), reveal discrepancies between targeted and measured W values. The systematically lower W content can likely be related with ICP preparation route and lower WO_3_ solubility in acids within the acid‐based digestion. Therefore, the following samples are labeled with the intended concentration of W (e.g., 1%_W).

The specific surface area is measured by gas adsorption and calculated according to Brunauer–Emmett–Teller (BET) theory. W‐containing CAMs have a higher BET surface area and can be related with decreased primary particle size. Though, the closer packed primary particles counteract further increase in BET surface area. Self‐synthesized CAMs have tendentially smaller surface areas, which is probably related to larger secondary particle sizes.

Rietveld refinements of the materials confirm an influence of W on the crystal structure (Table S1, Supporting Information). All refinements show a good fit (GOF < 2) between experimental and calculated values indicating validity of the results. The increased Li/Ni mixing disorder for W^6+^ incorporated NCM suggests an oxidation state balance for reasons of charge neutrality, i.e., respective reduction of Ni^3+^ to Ni^2+^. The similar ionic radii of Ni^2+^ and Li^+^ enhances the risk of Li/Ni mixing. In addition, an increase of unit cell volume is observed (Table S1, Supporting Information), which is in line with literature.^[^ ^12^ ^]^

**Figure 5:**
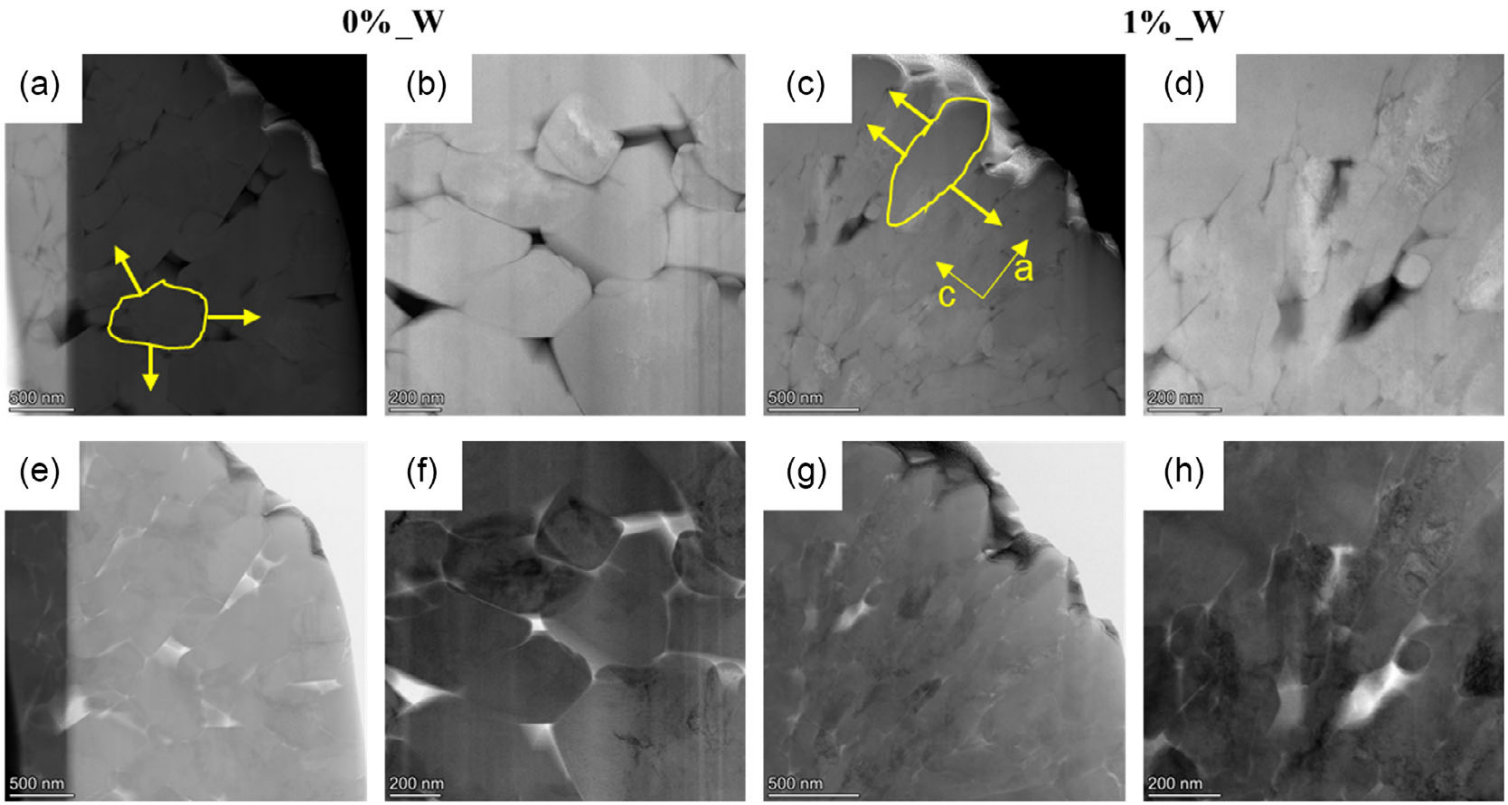
HAADF and BF‐STEM images of the CAMs showing the influence of W doping on the primary particle size: HAADF‐STEM images of a,b) 0%_W and c,d) 1%_W. BF‐STEM images of e,f) 0%_W and g,h) 1%_W. Schematic illustration points to a randomly distributed anisotropic strain of 0%_W, while the strain can be suggested to be more orientated in c‐direction for 1%_W.

High‐angle annular dark‐field (HAADF) and bright field scanning transition electron microscopy (BF‐STEM) images confirm grain‐like primary particles for W‐free NCM with relatively low particle density (**Figure** **5**a,b,e,f). The W‐containing CAM consists of smaller, elongated, and denser packed primary particles, which are orientated toward the particle surface (Figure 5c,d,g,h). Due to this particle design, anisotropic lattice stress can be better compensated in c‐direction, leading probably to the prevention of microcracks and therefore prolongation in cycle life via avoiding further rise in surface area, i.e., more “fresh surfaces” leading to additional failure cascades like phase changes, transition metal dissolution, and electrode crosstalk.^[^ ^14^, ^28^ ^]^

**Figure 6:**
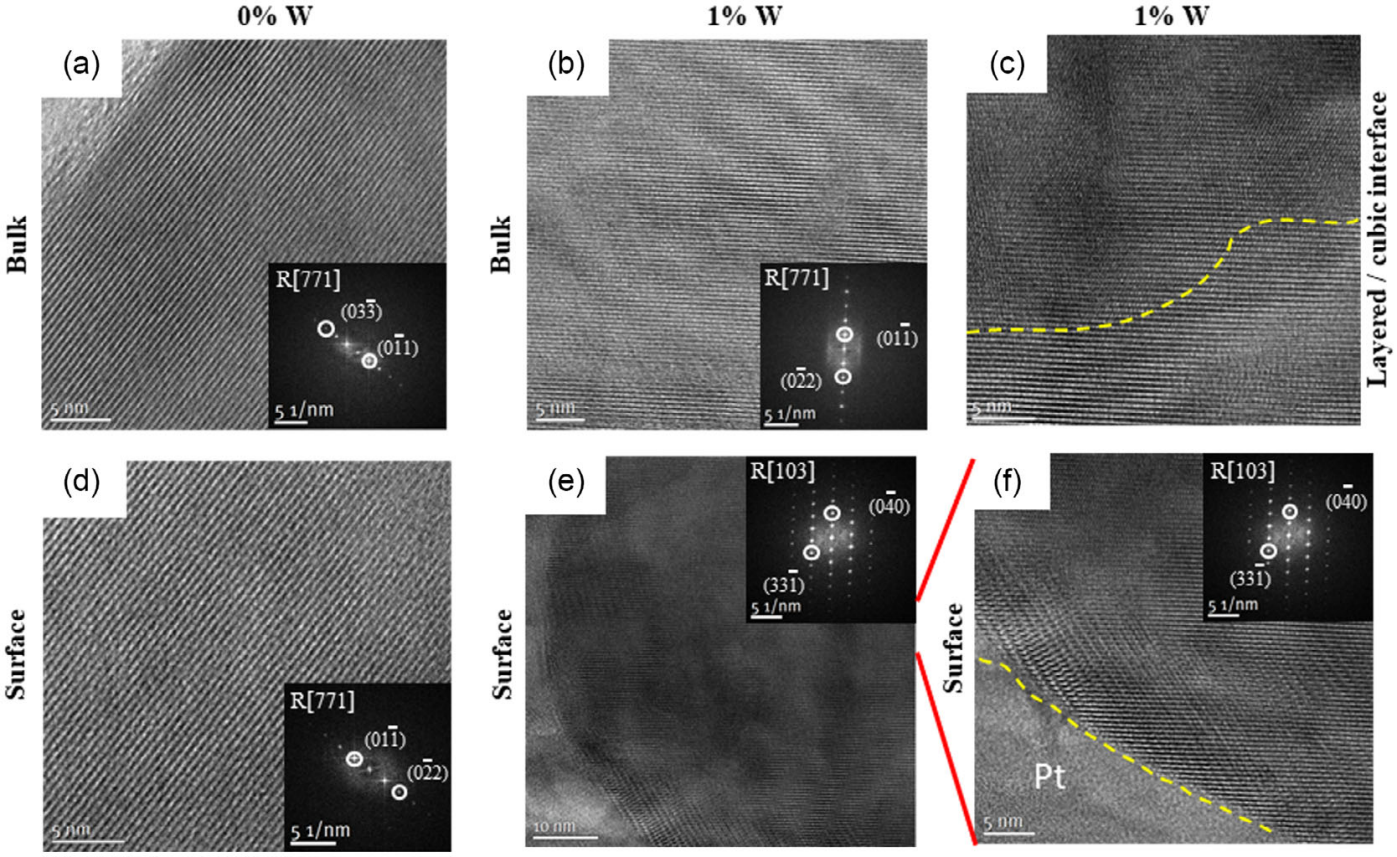
HR‐TEM images of a) W‐free and b) W‐containing NCM bulk and c) interface between cubic and layered phases of W‐containing CAM. The dashed line indicates transition from layered to cubic phases. HR‐TEM images of d) W‐free and e) W‐containing NCM surface f) magnified image of W‐containing surface. The inlets indicate alteration of crystal structure via W modification of surface from layered‐ to spinel/rock salt‐like phases. Pt coating is applied to reduce charging effects during TEM measurement.

High‐resolution (HR) TEM images of the reference material (**Figure** **6**) show layered structures ($R\bar{3}m$) of bulk, which is in line with XRD (Figure 4). While the surface of W‐free NCM also shows a layered structure, the W‐containing surface indicates a cubic “spinel‐like” (*Fd*3*m*)/rock salt (*Fm* $\bar{3}m$) phase. The thickness of this surface phase is ≈30 nm, which is in agreement with the literature.^[^ ^12^, ^29^ ^]^ Disordered phases with a thickness >20 nm are typically an indicator for electrochemically aged/“stressed” Ni‐rich layered oxides, which can impede Li transport.^[^ ^30^ ^]^ However, it is important to differentiate between theses “spinel‐like”/rock salt phases and the one related to W modification.^[^ ^12^, ^29^ ^]^

According to Zaker et al. W is located at the grain boundaries and near the surface of secondary particles after dry particle fusion and co‐precipitation.^[^ ^16^ ^]^ However, energy‐dispersive X‐ray spectroscopy (EDX) elemental mapping shows a homogeneous distribution of TMs on surface as well as inside the primary particles (Figure S5, Supporting Information), in line with Dalkilic et al.^[^ ^27^ ^]^

**Figure 7:**
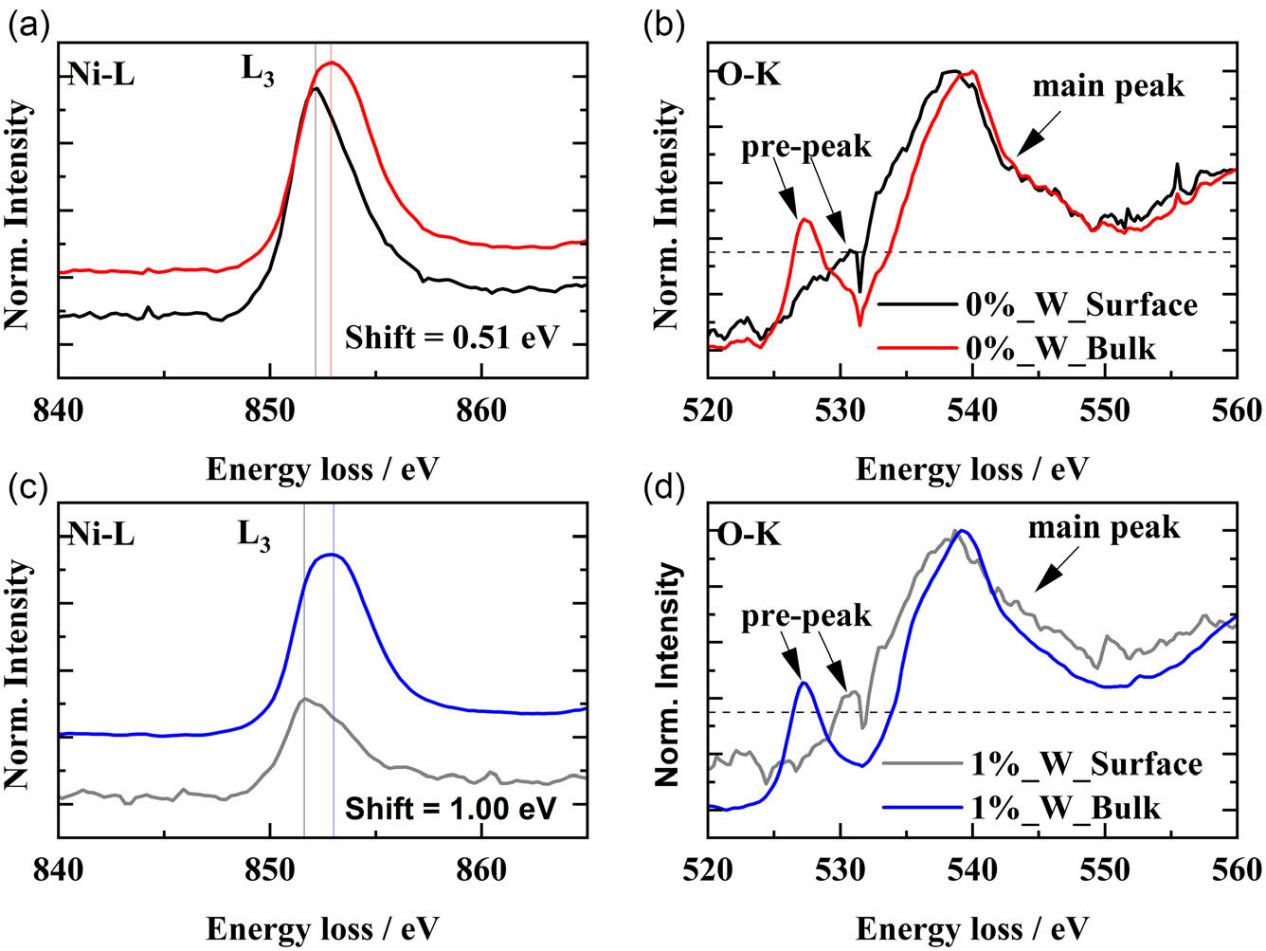
Magnified EELS data in a chosen energy loss area for W‐free NCM a) Ni‐L edge and b) O‐K edge and W‐containing NCM c) Ni‐L edge and d) O‐K edge. EELS spectra integrated from the surface and bulk of a secondary particle. Larger shift of the L_3_ peak and decrease in O‐K pre‐peak between surface and bulk indicates, respectively, pronounced formation of Ni^2+^and a W‐based phase, respectively.

HAADF‐STEM images and acquisition areas for electron energy loss spectroscopy (EELS) are shown in Figure S6, Supporting Information. The corresponding EELS spectra are shown in Figure S7, Supporting Information, while magnified images are shown in **Figure** **7**. The W‐free NCM core‐loss EELS spectra of Ni are acquired from the surface and bulk of a primary particle (Figure 7a). The energy position of the Ni‐L_3_ peak shifts toward lower energies from the bulk to the surface, which indicates a reduction of the Ni oxidation state near the surface, probably related to surface reaction with ambient air.^[^ ^31^, ^32^, ^33^ ^]^ Moreover, the O‐K edge pre‐peak relates to O 2p states and are hybridized with transition metal 3d states. The intensity and energy difference between the pre and main peak also reflects the oxidation state and coordination change of transition metal ions.^[^ ^34^, ^35^ ^]^ The intensity as well as the energy difference of the O‐K edge pre‐peak is lower at the surface of W‐free NCM compared to the bulk additionally confirming reduction of Ni oxidation states (Figure 7b).^[^ ^36^ ^]^ EELS analysis of the W‐containing CAM reveals a higher chemical shift of the L_3_ peak from the bulk to the surface compared to W‐free NCM (Figure 7c) and indicates enhanced Ni^2+^. The O‐K edge spectrum of the W‐containing surface shows also the pre‐peak with higher intensity as well as a different shape compared to W‐free NCM which is attributed to alterations in oxygen coordination, likely due to evolved amorphous W‐containing phases, e.g., Li_*x*_W_*y*_O_2_.^[^ ^11^ ^]^

The comparison between sol–gel coating and precipitation shows performance influence by both the dopant and the method of its incorporation, implying the complexity of CAM modification. Nevertheless, the beneficial impact of W can be related with surface modification, as indicated by SEM, TEM, and EELS analyses.

During calcination, highly charged W^6+^ ions are suggested to diffuse to the surface of the primary particles to form Li_*x*_W_*y*_O_*2*_ phases, while addition of W at the end of the precipitation process (as in 0.5%_W_CTR_Shell) or as a coating on the precursor (as in 0.5%_W and 1%_W) is particularly beneficial as the respective diffusion distance is low, thus leading to accumulation at the surface during lithiation, consequently enhancing the electrochemical aspects of the CAMs.

## 3 Conclusion

To further raise gravimetric/volumetric energies of Li ion batteries, the Ni content in layered oxide CAMs can be further increased even above 80%, in this work to 90%, i.e., LiNi_0.90_Co_0.06_Mn_0.04_O_2_ (NCM90‐6‐4), while structural issues remain and/or even enhance. Among many strategies, W doping/coating in aqueous media is promising as it can beneficially modify the CAM surface. Among different industrial‐applicable W incorporation strategies, the incorporation to precursor (pCAM) is reasonable as it is shown to enhance pCAM coverage and modifies primary particles while the promising direct W incorporation during pCAM synthesis in CTR is shown to have less effect and demonstrates the complexity of conditions during W incorporation and needs to be clarified in future works.

**Figure 8:**
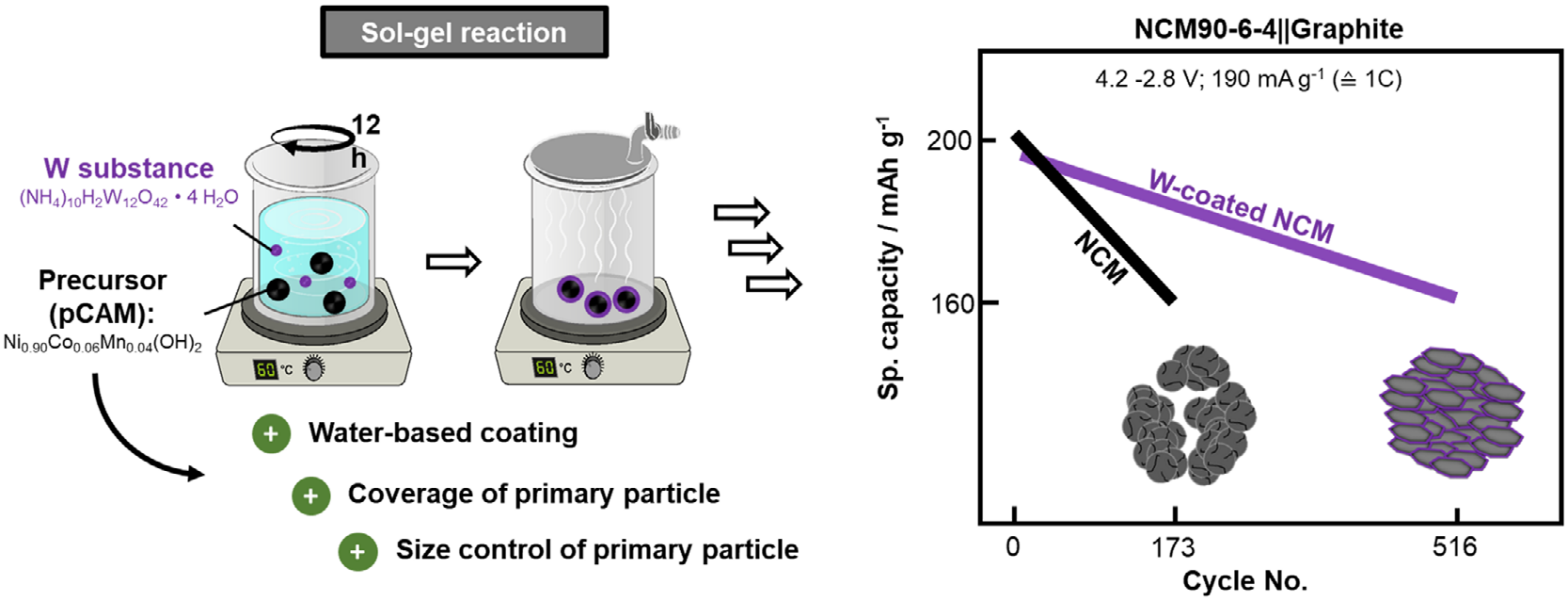
The coating of NCM90‐6‐4 can simply proceed within a sol–gel reaction utilizing W‐based substances and the (hydroxide‐based) pCAM, which allows water as processing solvent. Here, the W‐coating not only modifies particle surface but also elongates and decrease the subsequent particle shape and size within the final NCM after calcination. Finally, these particle alterations are suggested to enhance the cycle life toward >500 cycles for 80% SOH.

Though, the highly oxidized W^6+^ leads to a respective Ni^3+^ to Ni^2+^ reduction, enhances Li^+^/Ni^2+^ mixing, and decreases initial capacity, it improves the cycle life and rate capability, also at higher charge cutoff voltages (up to 4.5 V) and achieves even >500 cycles in NCM90‐6‐4||graphite cells at ≈80% SOH (**Figure** **8**).

As seen via SEM, W affects the morphology, i.e., elongates the primary particles, which is confirmed in HAADF and BF‐STEM images, and decreases their size compared to grain‐like reference (W‐free) CAMs. The anisotropic lattice stress is suggested to be better compensated in c‐direction, suppress microcracks, and avoid further rise in surface area, i.e., more “fresh surfaces,” thus additional failure cascades like phase changes, transition metal dissolution, and electrode crosstalk.

According to EELS analysis, the surface of W‐modified CAM tends to have enhanced Ni^2+^ and a lithiated W‐based phase, which can be related with spinel‐rock salt structures observed via HR‐TEM images of the surface.

Though, W incorporation appears promising and beneficial, more in‐depth studies are necessary in order to separate and evaluate the effects of changed morphology and surface crystal structure with electrochemical performance.

## 4 Experimental Section

4.1

4.1.1

##### Material Synthesis

The co‐precipitation of the Ni‐rich cathode precursor was carried out using a CTR (LCTR‐Tera 3300, Laminar, Rep. of Korea).^[^ ^23^ ^]^ The reactants NiSO_4_·6 H_2_O (Alfa Aesar), CoSO_4_·7 H_2_O (Acros Organics), and MnSO_4_·H_2_O (Acros Organics) with the mol ratio 90:6:4 were dissolved in the appropriate amount of deionized water to obtain a 1.5 m solution. The W‐containing solution was prepared by dissolving 0.5 at% Na_2_WO_4_·2 H_2_O and 3.5 at% MnSO_4_ · H_2_O in the appropriate amount of water to obtain a 1.5 m solution, while the Ni and Co concentrations remained unchanged. For the in situ encapsulation of the NCM90‐6‐4 precursor with W, a 0.15 m solution of Na_2_WO_4_·2 H_2_O was prepared. For the co‐precipitation of the transition metal hydroxides, a 4.875 m NaOH (Acros Organics solution) and 6.78 m ammonia solutions (VWR) were used.

At the beginning of the co‐precipitation, the CTR was filled with deionized water. Afterward, the temperature and the rotational number of the reactor were set to 70 °C and 700 rpm, respectively. The volume rates of the educt solutions were constant during the co‐precipitation, leading to a transition metal (TM):NaOH and TM:NH_3_ ratio of 1:2 and 1:1, respectively. After 16 h (four times the mean residence time), the TM(OH)_2_ containing suspension was collected. This suspension was washed till the pH was 7, filtered, and dried overnight at 80 °C. For the synthesis of the precursor which was coated in situ with Na_2_WO_4_, the corresponding solution was added at the end of the horizontally aligned reactor (see Figure 1b).

Tungsten coating on commercial NCM90‐6‐4 precursor was prepared via a sol–gel approach. Therefore, 20 g of the precursor was mixed with 0.28 g ammonium tungstate ((NH_4_)_10_H_2_(W_2_O_7_)_6_) (Sigma‐Aldrich) to obtain a 0.5 wt% containing coating. The mixture was mixed for 12 h in de‐ionized water at 60 °C. Afterward, the powder was dried by evaporating the water (*Büchi* evaporator).

The resulting NCM‐precursor was mixed with 2 mol% excess LiOH·H_2_O (Fisher Chemical) to compensate for the lithium loss during annealing. The mixture was preheated in a tube furnace (RS80/750/13, Nabertherm GmbH) for 3 h at 500 °C and then annealed for 12 h at 750 °C. The synthesis was performed with a heating rate of 2 °C min^−1^ and in an oxygen atmosphere (10 L h^−1^). Afterward, the materials were mortared and stored in a dry room (dew point < −50 °C, relative humidity 0.16%).

##### Electrode Preparation and Characterization

The Ni‐rich positive electrodes contained 94 wt% active material, 3 wt% poly(vinylidene difluoride) (PVdF) as binder (Solef 5130, Solvay) and 3 wt% carbon black as conductive agent (Super C65, Imerys Graphite & Carbon). First, the binder was dissolved in N‐methyl‐2‐pyrrolidone (NMP, anhydrous, purity: 99.5%, Sigma‐Aldrich). Afterward, the conductive agent and CAM were added and the mixture was dispersed by a high‐energy disperser (Dissolver Dispermat LC30, VMAGetzmann GmbH) at a speed of 2000 rpm for 5 min followed by mixing for 30 min at 10 000 rpm and finally 2000 rpm for 5 min.

The prepared electrode pastes were coated with a doctor blade (*Zehntner GmbH*) and an automatic film applicator (*Sheen Instruments*) on Al foil (washed with ethanol, 20 μm, *Nippon foil*). The average active mass loading of the positive electrode was 5 mg cm^−2^ for investigations in NCM||Li cells and 12 mg cm^−2^ for NCM||graphite cells. The positive electrodes (P) were dried for 2 h at 80 °C and calendared. Afterward, electrodes were punched out with a diameter of 14 mm and dried in a Büchi B‐585 glass drying oven at 120 °C for 12 h under reduced pressure (<50 mbar).

The negative electrodes (N) were prepared by mixing 95 wt% commercial synthetic graphite, 1.5 wt% styrene‐butadiene rubber (SBR, SB5521, LIPATON; *Polymer Latex GmbH*), and 3.0 wt% sodium‐carboxymethyl cellulose (Na‐CMC, Walocel CRT 2000 PPA12, *Dow Wolff Cellulosics*) as binders and 0.5 wt% carbon black (Super C65, *Imerys Graphite & Carbon*) as a conductive agent in de‐ionized water. The negative electrode paste was coated onto the copper foil (10 μm, *Nippon foil*), dried, and calendared to reach 30% porosity. The negative electrode (N) disks with a diameter of 15 mm and an average active mass loading of 7 mg cm^−2^ were punched out and dried in a Büchi B‐585 glass drying oven under reduced pressure (<50 mbar) at 120 °C for 12 h.

##### Cell Assembly and Electrochemical Characterization

The electrochemical investigations were performed in a two‐electrode configuration^[^ ^37^ ^]^ in coin cells (CR2032, *Hohsen*) with a polymer membrane (1‐layer, 16 mm Ø, Celgard 2500, *Celgard*) as separator. The separator was soaked with 35 μL of electrolyte 1 m LiPF_6_ in 3:7 vol% ethylene carbonate/ethyl methyl carbonate, EC/EMC, (*Solvionic*) with 2 wt% vinylene carbonate (VC, 99.9%). For NCM||Li metal cells, the prepared cathode was paired with Li metal foil (Ø15 mm, 500 μm in thickness; battery grade: purity ≥99.9%, *China Energy Lithium*). For NCM||graphite cells, the cathode was matched with a graphite anode, leading to a negative/positive capacity balancing ratio of N:P 1.15:1.00 (based on the 2nd cycle discharge capacity in NCM||Li and Li||C cells). The coin cells were assembled in a dry room atmosphere, and the reproducibility of the electrochemical data was verified by assembling three cells for each sample.

Electrochemical evaluation was performed *via* constant current (CC) charge–discharge cycling on a Maccor Series 4000 battery tester (*Maccor, Inc*.) at 20 °C. The rate capability, as well as the stability at higher cutoff voltages, was first investigated in NCM||Li metal cells. A specific current of 190 mA g^−1^ was defined as 1 C. First, the cells were rested for 6 h at open‐circuit voltage (OCV) to allow the wetting of the electrodes and separator. Afterward, the cells were cycled for two formation cycles at 0.1 C, three cycles at 0.2 C, and each five cycles at 0.33, 0.5, 1, and 3 C. To prevent Li metal plating on the negative electrode, the cells were always charged at a constant rate of 0.2 C while the discharged rate ranged between 0.1 and 3 C. The cell voltage window up to this point was between 4.3 and 2.9 V. After the rate capability investigations, the cells were cycled two cycles at 0.1 C followed by 15 cycles each at 0.33C up to 4.3, 4.4, and 4.5 V as UCVs.

The long‐term cycling was evaluated in NCM||graphite full cells in an operating voltage window between 4.2 and 2.8 V. Full cells were charged to 1.5 V for 15 min prior to 6 h at OCV to prevent Cu current collector corrosion.^[^ ^38^ ^]^ Cells were then cycled four cycles at 0.1 C to allow the formation of the solid electrolyte interphase as well as the cathode–electrolyte interphase. Afterward, the cells were cycled at 0.33 C till the capacity reaches 80% SoH (calculated based on the 5th cycle discharge capacity) with two regeneration cycles at 0.1 C every 100 cycles. After each charging step, a constant voltage (CV) step was performed with the limiting conditions of either achieving a time limit of a maximum 30 min or when the specific current reaches values below 0.05 C.

##### Characterization of the Cathode Materials

The morphology and surface of the prepared CAMs and electrodes were investigated via SEM using a Carl Zeiss AURIGA field emission microscope with an acceleration voltage of 3 kV and a working distance of 4 mm. FIB‐SEM was performed with a milling current of 2 nA and an acceleration voltage of 30 kV.

The particle sizes of the materials were measured with a Bettersize (3 P Instruments), which was based on static laser diffraction.

The stoichiometry of the CAMs was determined by ICP‐OES. Measurements were performed using an ARCOS (Spectro Analytical Instruments GmbH) with an axial‐positioned plasma torch. For analysis, the following emission lines were observed: Li, Ni, Co, Mn, and W. Measurement and digestion conditions were applied according to Evertz et al.^[^ ^39^ ^]^

For the measurement of the specific surface area, the powders were dried overnight at 200 ºC to remove adsorbed water under reduced pressure with a VacPrep 061 (*Micromeritics GmbH*). Afterward, the specific surface area was determined with BET calculation using krypton adsorption on a TriFlex (*Micromeritics GmbH).*

Powder XRD (Bruker D8 Advance) was performed between 10° and 90° at a step size of 0.02° s^−1^ using Cu‐K_α_ radiation (λ = 0.154 nm) at 40 kV and 20 mA with a divergence slit of 0.6 mm. The diffractograms were Rietveld‐refined based on a hexagonal *α*‐NaFeO_2_ structure with a space group $R\bar{3}m$ using Topas. For the refinements, oxygen was assumed to occupy 6c sites. Li was assumed to occupy 3a sites, and transition metals (TMs) were assumed to occupy 3b sites. The occupation of Ni^2+^ in the lithium layer was also quantified to account for the Li/Ni cation mixing disorder.

The HR‐TEM, HAADF‐STEM, BF‐STEM, EDX‐STEM, and EELS‐STEM techniques were carried out using a FEI Titan Themis G3 300 TEM equipped with a monochromator, a Gatan Image Filter (GIF) quantum ER/965P spectrometer, Super‐X EDX detector, Ceta 16 m camera, HAADF, and BF image detectors. The EELS spectra were recorded with 0.25 eV/channel dispersion, and an EELS spectrum image (EELS‐SI) was used to acquire the summed spectra over the selected area of an electrode. A dual‐EELS mode was used for acquiring low‐loss and core‐loss EELS spectra, and the energy of EELS spectra was calibrated using the zero‐loss peak. The background of EELS spectra was removed using the power law. An accelerating voltage of 300 kV was used for all TEM measurements. Since the electrode materials were beam‐sensitive, low electron beam current and low exposure time were used for acquiring EDX mapping and EELS‐SI. The TEM samples (lamella) were prepared using a FIB—SEM (Zeiss cross‐beam 340 FIB‐SEM). The TEM samples were transferred from glove box to TEM using a vacuum transfer holder (Gatan).

## Conflict of Interest

The authors declare no conflict of interest.

## Supporting information

Supplementary Material

## Data Availability

The data that support the findings of this study are available from the corresponding author upon reasonable request.
