# Supplementary material for: Ultrahigh Ni‐Rich (90%) Layered Oxide‐Based Cathode Active Materials: The Advantages of Tungsten (W) Incorporation in the Precursor Cathode Active Material
Source: Small Sci. 2024 Jul 10;4(10):2400135. doi: 10.1002/smsc.202400135 (PMC11935202; doi:10.1002/smsc.202400135)
Supplement: Supplementary file 1 — Supplementary Material [file SMSC-4-2400135-s001.pdf]

Supporting information

Ultra High Ni-Rich (90%) Layered Oxide-based Cathode Active Materials (CAMs): The Advantages of Tungsten (W) Incorporation in the Precursor CAM

Marcel Heidbüchel[1], Dr. Aurora Gomez-Martin,[1], Lars Frankenstein[1], Ardavan Makvandi[3], Dr. Martin Peterlechner[3], Prof. Dr. Gerhard Wilde[3], Prof. Dr. Martin Winter[1,2], Dr. Johannes Kasnatscheew[1,]*

[1]University of Münster, MEET Battery Research Center, Institute of Physical Chemistry, Corrensstr. 46, 48149 Münster, Germany

[2] Helmholtz Institute Münster, IEK-12, Forschungszentrum Jülich GmbH, Corrensstr. 46, 48149 Münster, Germany

[3]University of Münster, Institute of Materials Physics, Wilhelm-Klemm-Str.10, 48149 Münster, Germany

*Corresponding authors: johannes.kasnatscheew@uni-muenster.de (J. Kasnatscheew),

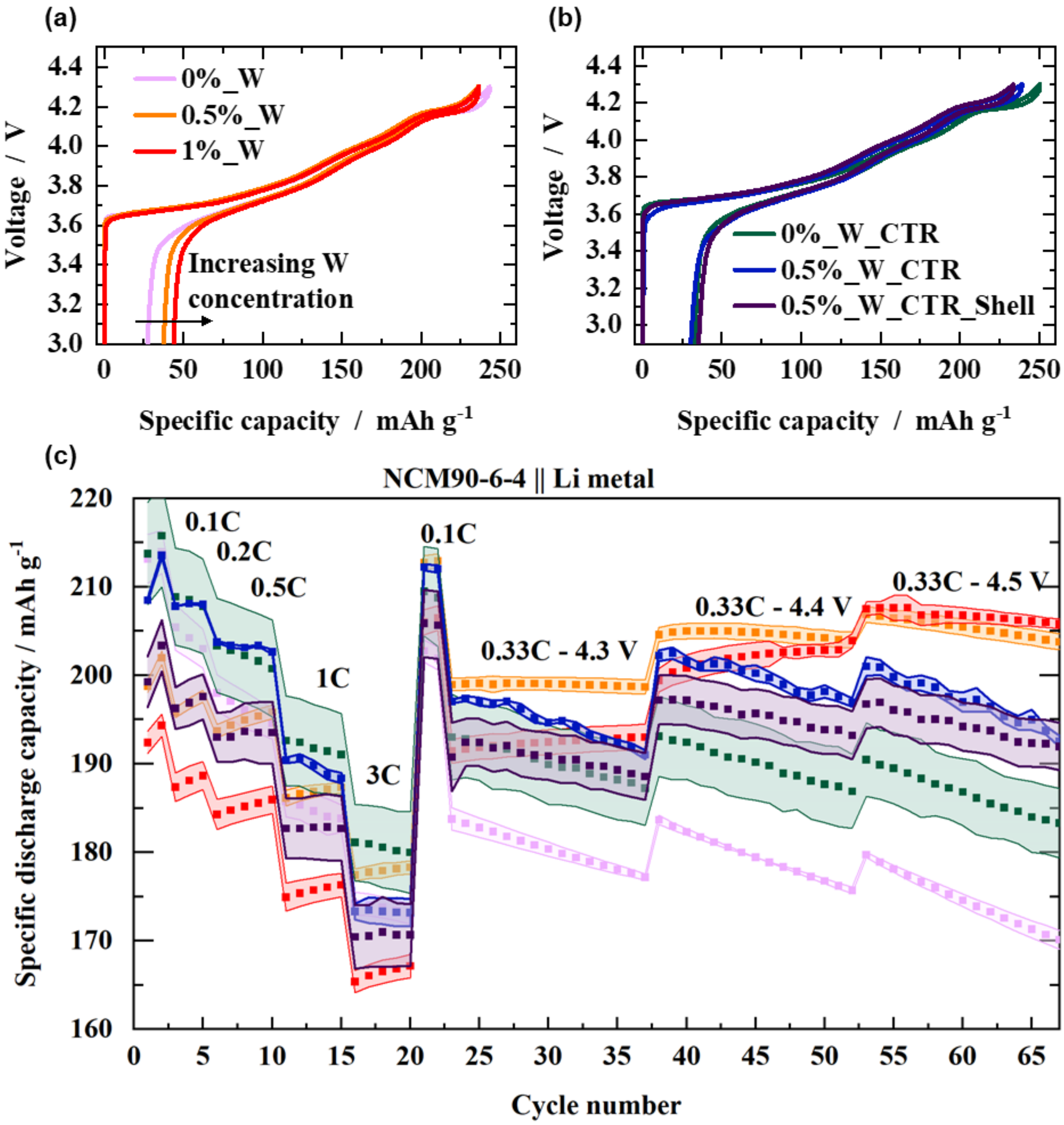

Figure S1: Galvanostatic charge/discharge data in NCM||Li cells. (a) First cycle voltage profile of CAMs based on commercial precursor. (b) First cycle voltage profile of self-synthesized materials. (c) Specific discharge capacity in NCM90-6-4 || Li metal cells (two-electrode configuration) in a cell voltage range of 4.3-2.9 V, 4.4-2.9 V or 4.5-2.9 V (1C=190 mA g^{-1}); Electrolyte: 1M $LiPF_6$ in EC:EMC 3:7 + 2 wt.%VC. Error bars represent the standard deviation of three cells per type of sample. W modification decreases the initial discharge capacity and initial C_{eff}, but stabilizes the capacity at elevated voltages.

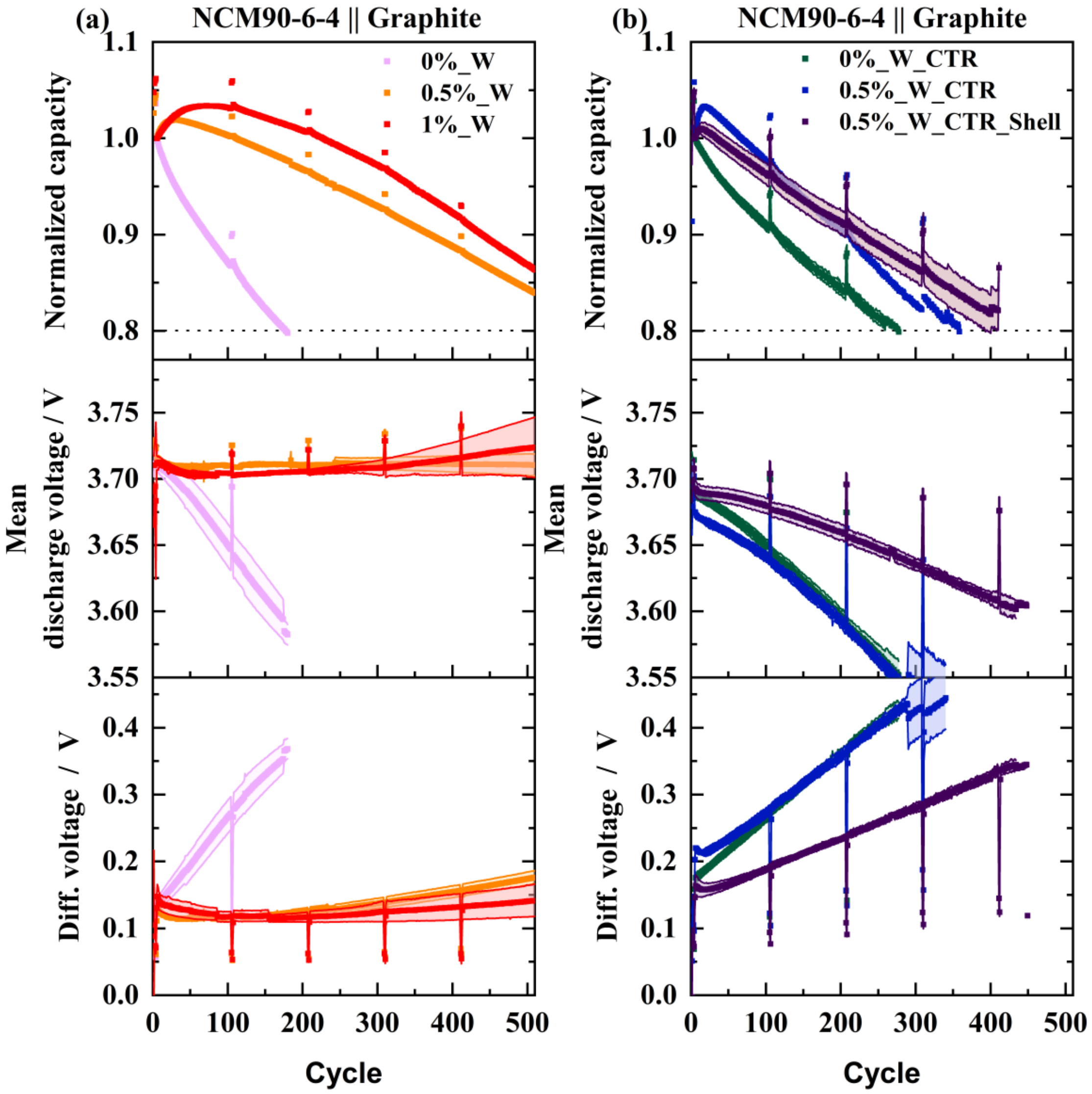

Figure S2:Galvanostatic charge/discharge cycling data from NCM||Graphite cells, with (a) CAM from sol-gel coating (b) CAM from CTR. W-containing NCM increase cycle life and decrease voltage hysteresis. Electrolyte: 1M $LiPF_6$ in EC:EMC 3:7 + 2 wt.%VC. Error bars represent the standard deviation of three cells per type of sample.

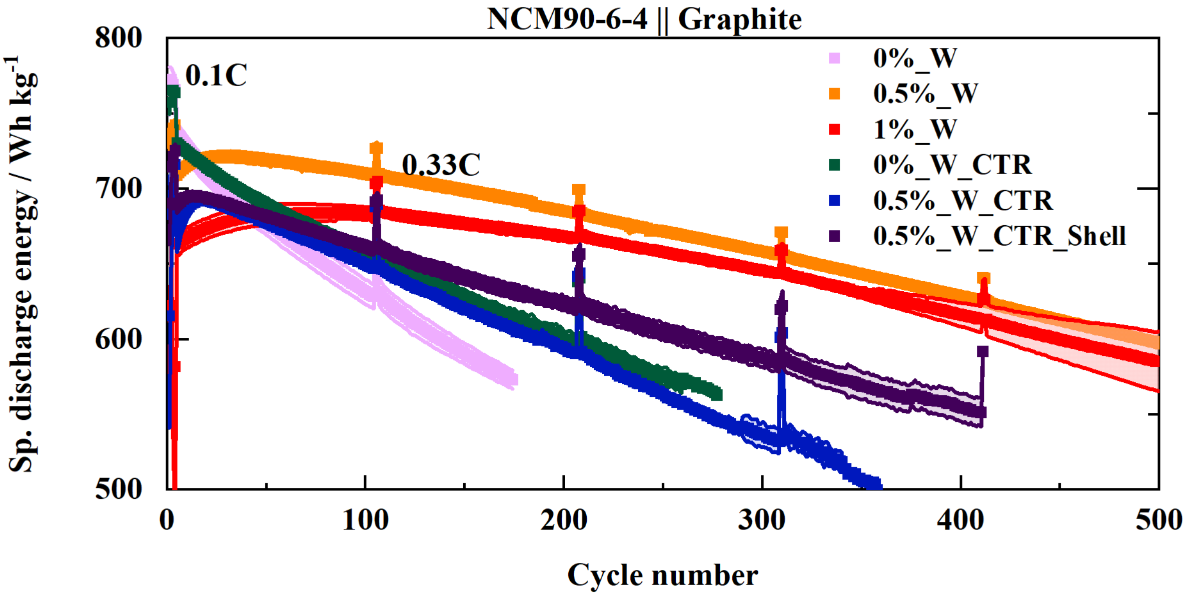

Figure S3: Galvanostatic charge/discharge cycling data from NCM||Graphite cells. Specific discharge energies indicate benefit of sol-gel modified NCM. Specific discharge energies on material level of the long-term cycling stability experiments in NCM90-6-4|| graphite full-cells at a rate of 0.33C. Standard deviation was calculated from at least three different cells. Cell voltage range: 4.2 – 2.8 V, N/P-ratio: 1.15:1.00, electrolyte: 1 M $LiPF_6$ in 3:7 vol. % EC/EMC +2 wt.% VC.

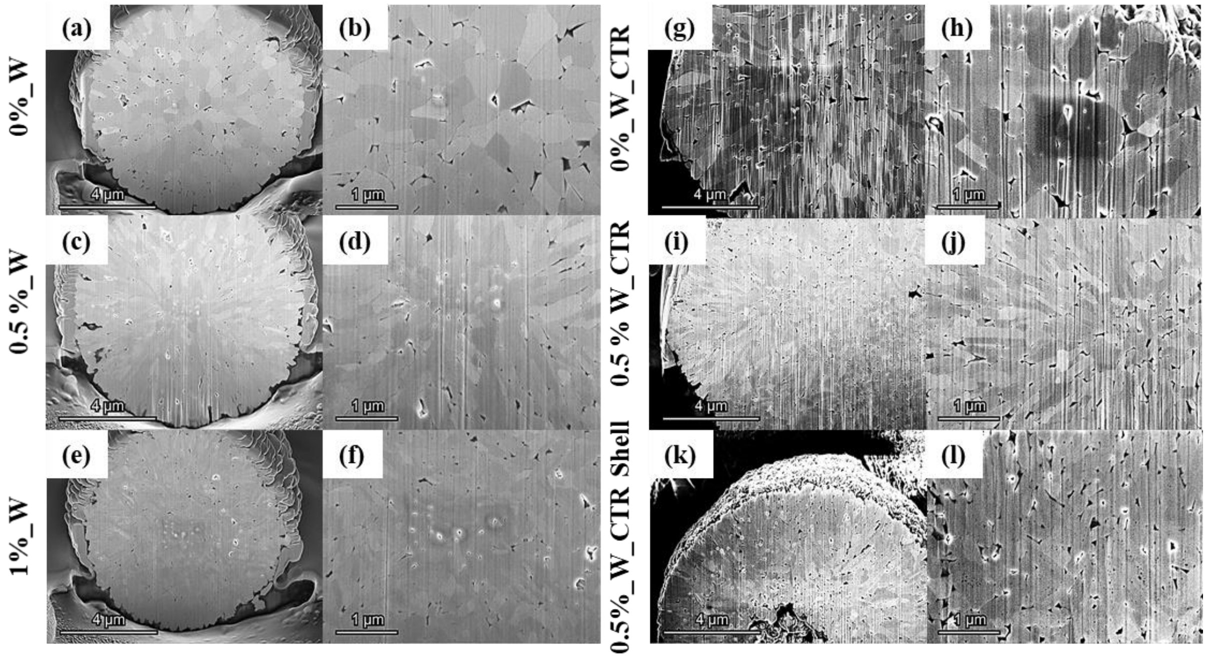

Figure S4: FIB-SEM cross-sectional view of the different CAMs indicating smaller primary particles upon W modification (a,b) NCM90-6-4 reference without W addition (c,d) 0.5%_W (e,f) 1%_W (g,h) NCM90-6-4 reference synthesized in CTR (i,j) 0.5%_W_CTR synthesized in CTR (k,l) 0.5%_W_CTR_Shell synthesized in CTR with *in situ* coating of Na_2WO_4.

Table S1: Rietveld refinement data for XRD powder analyses of pristine CAMs.: R-weighted pattern (R_{wp}), R-expected (R_{exp}), goodness of fit (GOF), Crystallite size, lattice parameters a and c, unit cell volume (V), Li/Ni mixing.

Material	0%_W	0.5%_W	1%_W	0%_W_CTR	0.5%_W_CTR	0.5%_W_CTR_Shell
R_{wp}	2.604	2.064	2.019	1.323	1.412	1.349
R_{exp}	1.676	1.671	1.684	1.110	1.176	1.132
GOF	1.553	1.235	1.199	1.192	1.200	1.192
Cry- size / nm	303.556	148.562	80.941	495.831	110.503	210.223
a /10^{-10} m	2.880	2.873	2.872	2.874	2.876	2.875
c /10^{-10} m	14.214	14.185	14.178	14.203	14.202	14.197
V / 10^{-30} m^3	102.071	101.390	101.303	101.590	101.705	101.621
Li / Ni mixing %	2.767	3.496	6.333	3.226	5.673	6.076

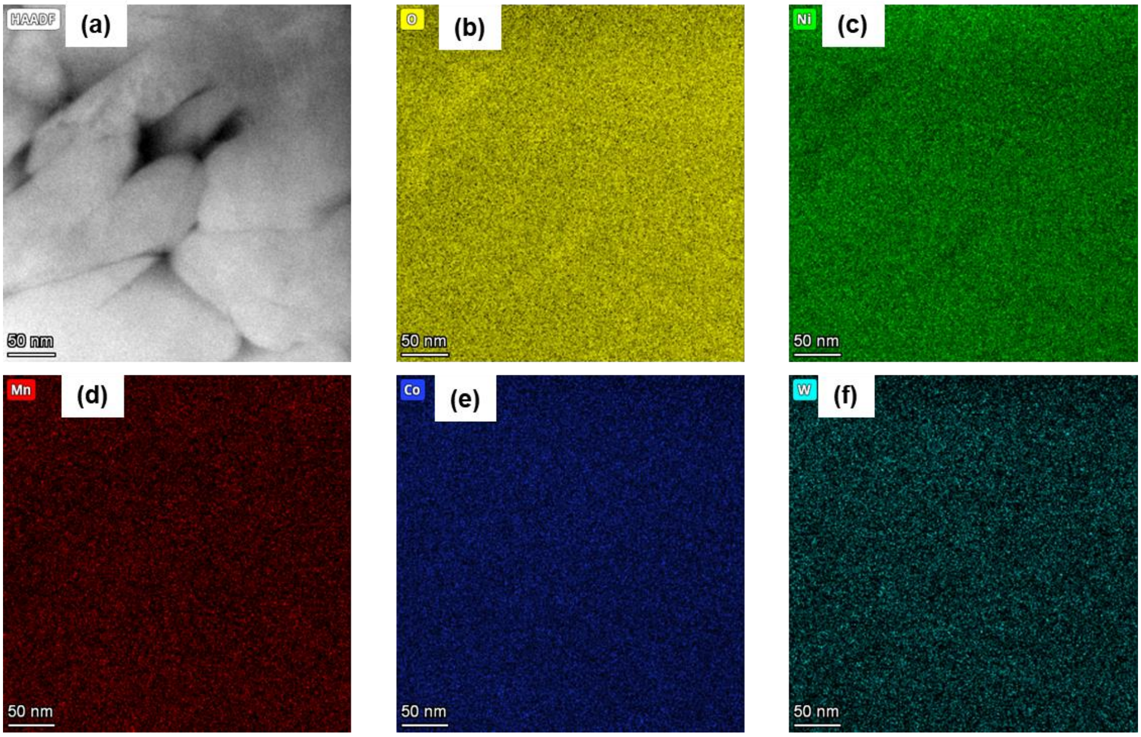

Figure S5: (a) HAADF-STEM image and (b-f) EDX elemental mapping of 1%_W CAMs indicating homogeneous W distribution.

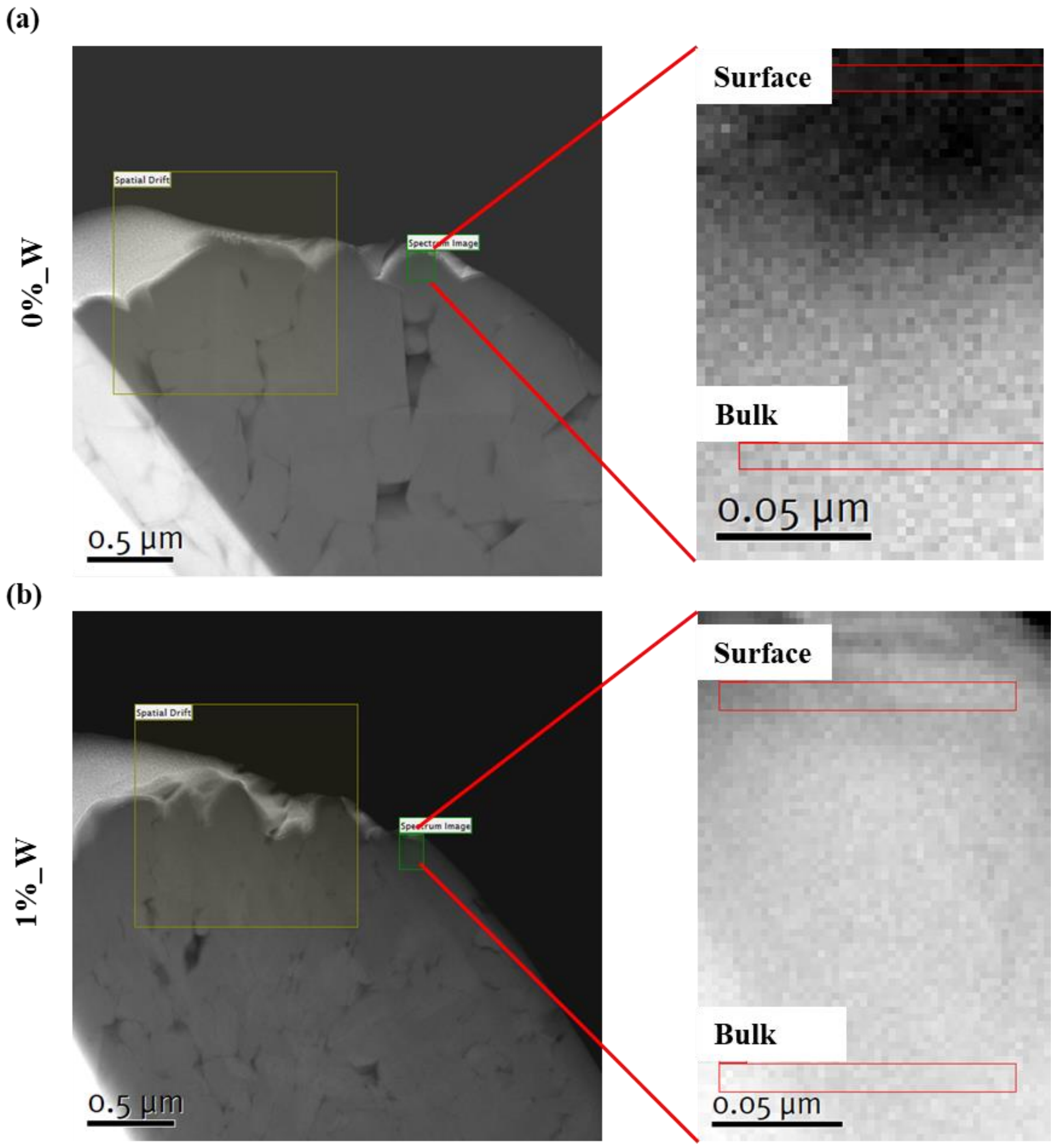

Figure S6: (a) HAADF-STEM image, core-loss EELS – SI acquired from the small rectangle for 0%_W CAM (b) HAADF-STEM image, core-loss EELS – SI acquired from the small rectangle for 1%_W CAM.

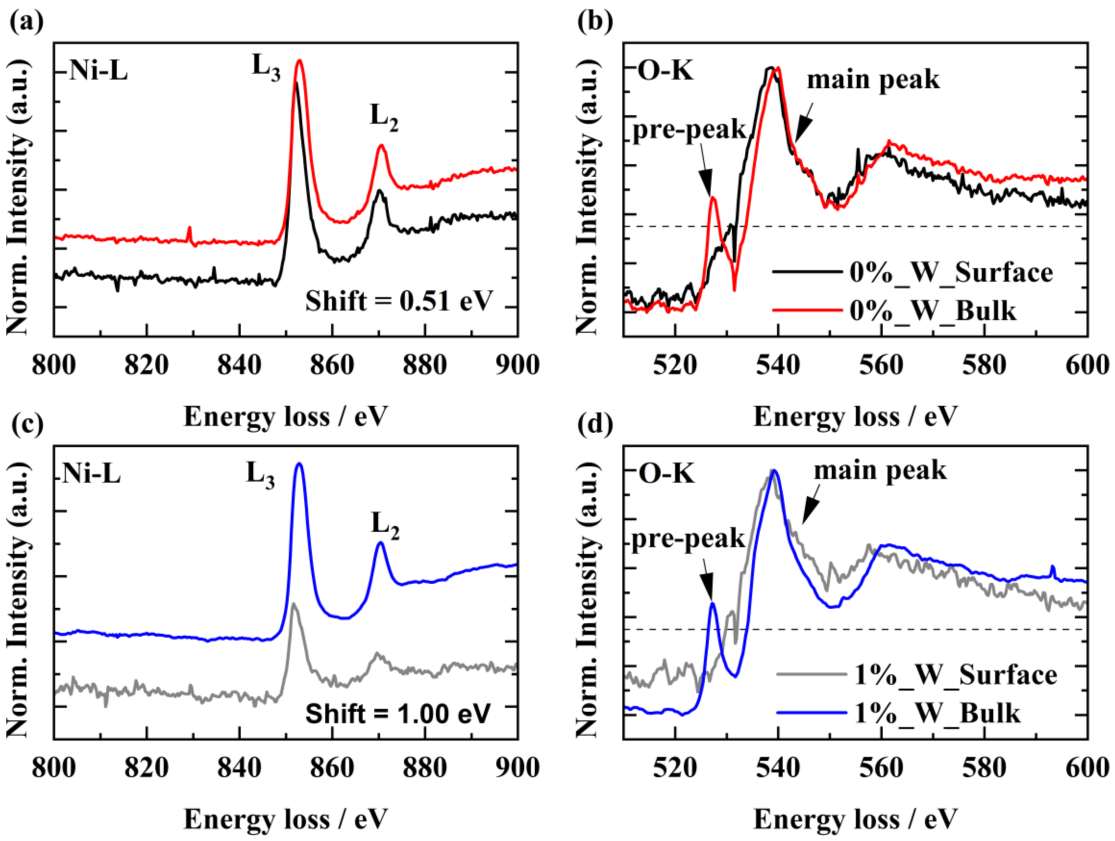

Figure S7: EELS data for W-free NCM (a) Ni-L edge, and (b) O-K edge and W-containing NCM (c) Ni-L edge, and (d) O-K edge. EELS spectra integrated from the surface and bulk of a secondary particle. Larger shift of the L_3 peak and decrease in O-K pre-peak between surface and bulk indicates, respectively, pronounced formation of $Ni^{2+}$and a W-based phase, respectively.
